# The Crucial Role of PPARγ-Egr-1-Pro-Inflammatory Mediators Axis in IgG Immune Complex-Induced Acute Lung Injury

**DOI:** 10.3389/fimmu.2021.634889

**Published:** 2021-02-25

**Authors:** Chunguang Yan, Jing Chen, Yue Ding, Zetian Zhou, Bingyu Li, Chunmin Deng, Dong Yuan, Qi Zhang, Ximo Wang

**Affiliations:** ^1^Department of Pathogenic Biology and Immunology, Medical School of Southeast University, Nanjing, China; ^2^Jiangsu Provincial Key Laboratory of Critical Care Medicine, Zhongda Hospital of Southeast University, Nanjing, China; ^3^Tianjin Key Laboratory of Acute Abdomen Disease Associated Organ Injury and Integrated Chinese and Western Medicine (ITCWM) Repair, Institute of Integrative Medicine for Acute Abdominal Diseases, Integrated Chinese and Western Medicine Hospital, Tianjin University, Tianjin, China; ^4^Emergency Department, Jintan Hospital, Jiangsu University, Changzhou, China

**Keywords:** acute lung injury, PPARγ, Egr-1, pro-inflammatory mediators, inflammation

## Abstract

**Background:**

The ligand-activated transcription factor peroxisome proliferator-activated receptor (PPAR) γ plays crucial roles in diverse biological processes including cellular metabolism, differentiation, development, and immune response. However, during IgG immune complex (IgG-IC)-induced acute lung inflammation, its expression and function in the pulmonary tissue remains unknown.

**Objectives:**

The study is designed to determine the effect of PPARγ on IgG-IC-triggered acute lung inflammation, and the underlying mechanisms, which might provide theoretical basis for therapy of acute lung inflammation.

**Setting:**

Department of Pathogenic Biology and Immunology, Medical School of Southeast University

**Subjects:**

Mice with down-regulated/up-regulated PPARγ activity or down-regulation of Early growth response protein 1 (Egr-1) expression, and the corresponding controls.

**Interventions:**

Acute lung inflammation is induced in the mice by airway deposition of IgG-IC. Activation of PPARγ is achieved by using its agonist Rosiglitazone or adenoviral vectors that could mediate overexpression of PPARγ. PPARγ activity is suppressed by application of its antagonist GW9662 or shRNA. Egr-1 expression is down-regulated by using the gene specific shRNA.

**Measures and Main Results:**

We find that during IgG-IC-induced acute lung inflammation, PPARγ expression at both RNA and protein levels is repressed, which is consistent with the results obtained from macrophages treated with IgG-IC. Furthermore, both *in vivo* and *in vitro* data show that PPARγ activation reduces IgG-IC-mediated pro-inflammatory mediators’ production, thereby alleviating lung injury. In terms of mechanism, we observe that the generation of Egr-1 elicited by IgG-IC is inhibited by PPARγ. As an important transcription factor, Egr-1 transcription is substantially increased by IgG-IC in both *in vivo* and *in vitro* studies, leading to augmented protein expression, thus amplifying IgG-IC-triggered expressions of inflammatory factors *via* association with their promoters.

**Conclusion:**

During IgG-IC-stimulated acute lung inflammation, PPARγ activation can relieve the inflammatory response by suppressing the expression of its downstream target Egr-1 that directly binds to the promoter regions of several inflammation-associated genes. Therefore, regulation of PPARγ-Egr-1-pro-inflammatory mediators axis by PPARγ agonist Rosiglitazone may represent a novel strategy for blockade of acute lung injury.

## Background

Excessive formation of IgG immune complexes *in vivo* has been demonstrated to be associated with a variety of diseases, such as systemic lupus erythematosus, rheumatoid arthritis, acute glomerulonephritis, mixed cryoglobulinemia and vasculitis (1–7). Moreover, the pulmonary diseases, such as acute lung injury (ALI) and its more severe form—acute respiratory distress syndrome (ARDS), are also reported to be related with deposition of immune complexes in the tissue (8, 9). During IgG-IC induction of ALI/ARDS, the local macrophages are firstly activated through engagement of Fcγ receptors with the ligands, resulting in the release of numerous cytokines and chemokines. Then, under the action of the inflammatory factors, alveolar epithelial cells are activated and neutrophils are recruited into the pulmonary tissue. Finally, acute lung injury is triggered by the severe inflammatory response. However, the molecular events responsible for productions of inflammation-associated mediators are poorly understood.

PPARγ is a type II nuclear receptor, which is rich in adipose tissue. Actually, other types of cells and tissues also generate PPARγ (10–12). A large number of studies focus on its roles in regulation of gene expressions associated with lipid uptake and adipogenesis, and find that PPARγ is involved in a broad spectrum of lipid metabolism disorder-mediated diseases, such as obesity, diabetes and atherosclerosis (13–15). However, the importance of PPARγ in immune regulations has also been recognized. For example, PPARγ is required for IL-9 production in T_H_2 cells, which might exert regulatory effects on acute allergic skin inflammatory response in human beings (16). Reddy et al. report that PPARγ activation has protective effects against cigarette smoke-stimulated inflammatory responses during chronic obstructive pulmonary disease (17). Except for its effect on chronic pulmonary disease, the role of PPARγ in acute lung injury has also been examined. There are data that ventilation- or LPS-induced acute lung injury is relieved by PPARγ, but the conclusions are obtained by using PPARγ antagonist or agonist (18–20). So, the nonspecific effects of the chemical reagents cannot be excluded. More importantly, the influence of PPARγ on IgG-IC-induced acute lung injury and the corresponding mechanism have not been elucidated.

Egr-1 also named as NGFI-A (nerve growth factor-induced protein A) is a nuclear protein acting as a transcription factor. Its expression can be induced by a variety of stimulus including cytokines, neurotransmitters and growth factors (21). However, the effect of IgG-IC treatment on its expression remains enigmatic. Aberrant Egr-1 expression or activity has been detected in various human diseases, such as atherosclerosis, cancer, ischemic injury and TGF-β-dependent pro-fibrotic responses (21). Notably, the role of Egr-1 in inflammatory responses has also been extensively investigated. For example, cigarette smoke-induced chemokine release from primary human lung fibroblasts is dependent on Egr-1 (22). In addition, the secretion of inflammatory cytokines induced by Mycoplasma pneumonia infection *in vitro* and *in vivo* is also positively regulated by Egr-1 (23). However, its influence on acute lung inflammation and the underlying mechanism after intrapulmonary deposition of IgG-IC remain mysterious.

Given the importance of PPARγ and Egr-1 in inflammatory responses, the present study intends to determine the roles of the two transcription factors in IgG-IC-induced acute lung injury, and the possible interaction between PPARγ and Egr-1. We find, for the first time, that PPARγ expression is downregulated, while Egr-1 expression is upregulated in IgG-IC-treated mouse lungs and macrophages. Furthermore, we prove that Egr-1 enhances IgG-IC-induced inflammatory response by directly binding to the promoter regions of the pro-inflammatory mediators, and PPARγ suppresses inflammation by inhibiting production of Egr-1. Taken together, the data show that during IgG-IC-induced acute lung injury, PPARγ may play an essential role in regulating inflammatory response by interfering with the expression of its downstream target Egr-1, and PPARγ agonist Rosiglitazone may be used clinically for blockade of acute lung injury in the future.

## Materials and Methods

### Animals, Cell Culture, and Reagents

Pathogen-free male C57BL/6 mice that are 8–12 weeks old are obtained from Model Animal Research Center of Nanjing University (Nanjing, China), and all animal experiments are performed according to the protocol approved by Southeast University. RAW264.7 cells and HEK293 cells are purchased from American Type Culture Collection and cultured in DMEM supplemented with 10% fetal bovine serum (Gibico). Bovine serum albumin (BSA) and rabbit anti-BSA IgG (α-BSA) are purchased from Invitrogen and MP Biomedicals, respectively. ELISA kit for mouse albumin is purchased from Bethyl Laboratories. ELISA kits for TNF-α, MCP-1, MIP-1α and MIP-2 are purchased from R&D Systems. PPARγ antagonist GW9662, PPARγ agonist Rosiglitazone (ROSI) and puromycin are purchased from Cayman Chemical.

### IgG-IC-Induced Acute Lung Injury and Analysis of Bronchoalveolar Lavage Fluids (BALFs)

Mice are anesthetized by intraperitoneal injection of 1.5% sodium pentobarbital. Then rabbit anti-BSA antibodies are administrated into mouse lungs through the airways. Immediately after the operation, the mice are treated by intravenous injection of BSA dissolved in PBS. Four hours later, whole lungs or bronchoalveolar lavage fluids (BALFs) are collected.

BALFs are centrifuged at 3,000 rpm for 5 min at 4°C, and then cell-free supernatants are collected and subjected to analyze albumin, TNF-α, MCP-1, MIP-1α and MIP-2 levels by ELISA. Albumin contents derived from IgG-IC-treated mice are divided by those from the corresponding control mice to obtain values of lung permeability indexes. The remaining cell pellets are resuspended in HBSS containing 0.5% BSA. Total white blood cells are measured with hemocytometer. Cytospin centrifugation is used for preparation of samples for cell differentials. Samples are fixed and stained with Diff-Quik Stain (Solarbio^®^ LIFE SCIENCES, China), which is followed by analysis of percentage of neutrophils.

### Construction of Expression Vectors and Promoter-Driven Reporters

Adenoviral vectors promoting ectopic expression of PPARγ are constructed by following the instruction provided by BD Biosciences. Briefly, PPARγ-encoding sequences (Accession Number NM_001127330) are amplified from mouse lung cDNA library, and the DNA sequences are ligated with the shuttle vector—pShuttle2, to form the recombinational plasmids—pShuttle2-PPARγ. Enzyme-digested pShuttle2-PPARγ is extracted and linked with the adenoviral vector. Then the recombinational adenovirus vectors are linearized by the restriction enzyme PacI, which is followed by transfection of the linear DNA into HEK293 cells to make the infectious adenovirus (Ad-PPARγ). The obtained Ad-PPARγ is stored at –80°C, and tittered by utilizing the titer kit from BD Biosciences before use.

Control plasmid is constructed by ligating the amplified EGFP encoding fragment from pIRES-EGFP (Clontech) with pcDNA3.1-Myc-His (Invitrogen). Egr-1-encoding sequences (Accession Number NM_007913) are cloned from mouse lung cDNA library by PCR. The recombinational plasmids expressing Egr-1 are then formed by ligating the amplified DNA sequences with pcDNA3.1-Myc-His.

We firstly generate double-stranded oligos containing the PPARγ or Egr-1 interference sequence, and then link them to the vector—miRZip™ shRNA Expression Lentivector (System Biosciences). The transfer plasmids together with two other plasmids—pMD2.G and psPAX2 (Addgene plasmids 12259 and 12260) are co-transfected into HEK293 cells by using Lipofectamine 2000 (Invitrogen) to make mature viral particles that are titered by measuring GFP positivity after transduction.

The full length of TNF-α (Accession Number NC_000083)/MCP-1 (Accession Number NC_000077)/MIP-1α (Accession Number) promoter region is amplified by using mouse genomic DNA as the template. Then TNF-α Luc, MCP-1 Luc and MIP-1α Luc are constructed by ligating their corresponding promoter fragments with pGL4-basic vector (Promega). Mutations are introduced to the Egr-1 binding sites in TNF-α, MCP-1 and MIP-1α promoter regions by using the Mut Express II Fast Mutagenesis Kit V2 (Vazyme, China). pRL-TK plasmids used as internal references are obtained from Promega.

### Airway Injection of Recombinational Viral Vectors

Ad-GFP (BD Biosciences) or Ad-PPARγ is intratracheally administrated into mice. Three days later, the infected mice are used for the subsequent experiments. Mice receiving airway administration of shRNA NC or Egr-1 shRNA are used 5 days after infection.

### Construction of Stable Cell Line

RAW264.7 cells are infected by control or PPARγ/Egr-1 shRNA lentiviral particles at a MOI of 30. Seventy-two hours later, the cells are treated with 3 μg/ml puromycin. The survival cells are selected, and the knockdown efficiency is confirmed by qPCR.

### Western Blot Analysis

Total cellular or tissue proteins are extracted by using radioimmune precipitation assay buffer from Beyotime Biotechnology (China). Then 30 μg of proteins are run on a 12% SDS-polyacrylamide gel and examined by using the following antibodies: goat anti-PPARγ (Santa Cruz, catalog number sc-1984X), mouse anti-Egr-1 (Santa Cruz, catalog number sc-515830), and mouse anti-GAPDH (Proteintech, catalog number 60004-1-Ig).

### Quantitative RT-PCR Assay

Total RNAs are extracted with Trizol (Invitrogen), and reverse transcribed into cDNAs using PrimeScript™ RT reagent Kit obtained from TaKaRa. qPCR assays are conducted with the following protocol: an initial step at 95°C for 3 min followed by 40 cycles of 95°C for 5 s and 60°C for 30 s. The primers used are as follows: PPARγ, 5′ primer, 5′-agg gcg atc ttg aca gga aa-3′, and 3′ primer, 5′-cga aac tgg cac cct tga aa-3′; Egr-1, 5′ primer, 5′-cga gtt atc cca gcc aaa cg-3′, and 3′ primer, 5′-gaa gac gat gaa gca gct gg-3′; TNF-α, 5′ primer, 5′-CGT CAG CCG ATT TGC TAT CT-3′, and 3′ primer, 5′-CGG ACT CCG CAA AGT CTA AG-3′; MCP-1, 5′ primer, 5′-AGG TCC CTG TCA TGC TTC TG-3′, and 3′ primer, 5′-TCT GGA CCC ATT CCT TCT TG-3′; MIP-1α, 5′ primer, 5′-ATG AAG GTC TCC ACC ACT GC-3′, and 3′ primer, 5′-CCC AGG TCT CTT TGG AGT CA-3′; GAPDH: 5′ primer, 5′-GCC TCG TCT CAT AGA CAA GAT G-3′, and 3′ primer, 5′-CAG TAG ACT CCA CGA CAT AC-3′.

### Measurement of Pulmonary Myeloperoxidase (MPO) Activity

Four hours after airway deposition of IgG-IC, mouse lungs are harvested and homogenized in the buffer containing the following components: 0.5% hexadecyltrimethylammonium bromide, 50 mM potassium phosphate buffer and 5 mM EDTA. Then the homogenates are subjected to sonication. Cell-free supernatants are mixed with the MPO analysis buffer consisted of 5 μg/ml H_2_O_2_, 100 mM potassium, and 167 μg/ml *o*-dianisidine dihydrochloride. MPO contents are determined by measuring the change in optical density (at 450 nm) per min using the 96-well plate reader.

### Luciferase Assays

Cells are cultured in 12-well plates. Twelve hours later, the cells are transfected with total of 0.5 μg plasmids using Fugene^®^6 Transfection Reagent (Promega) according to the manufacturer’s guideline. Twenty-four hours after transfection, cells are lysed and the lysates are subjected to measurement of luciferase activities with the Dual-Luciferase Reporter Assay System purchased from Promega.

### Statistical Assay

All data are represented as the mean ± S. E. M. Significance is indicated when *p* value is less than 0.05. Data sets are analyzed using Student’s t test or one-way ANOVA, with individual group means being compared with the Student-Newman-Keuls multiple comparison test.

## Results

### PPARγ Negatively Regulates IgG-IC-Induced Acute Lung Injury

The roles of PPARγ in pulmonary diseases have been extensively explored. However, during IgG-IC-stimulated acute lung injury, its expression and function have not been determined. So, we first examine if PPARγ production is changed in the mouse lung after intratracheal deposition of IgG-IC. As shown in **Figure S1A**, IgG-IC treatment leads to a 36% decrease in PPARγ production at mRNA level, which is consistent with the Western blot assay (**Figure S1B**).

**Figure 1:**
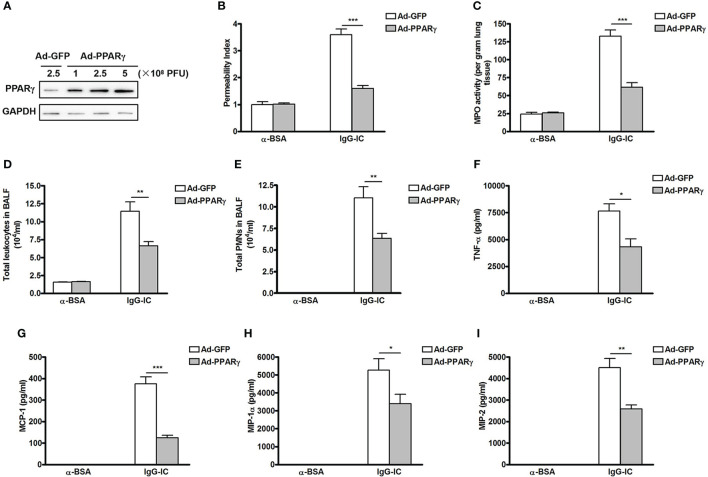
Ectopic expression of peroxisome proliferator-activated receptor (PPAR)γ in the lung inhibits IgG immune complex (IgG-IC)-induced acute lung injury. **(A)** Different doses of adenovirus are injected into lungs *via* airways. Seventy-two hours later, lungs are harvested and total proteins are extracted. Then Western blot assays are conducted by using antibodies recognizing PPARγ and GAPDH, respectively. Mice are treated by airway administration of 1 × 10^8^ PFU of Ad-GFP or Ad-PPARγ. Three days later, acute lung injury is induced by treating the mice with IgG-IC. Four hours later, bronchoalveolar lavage (BAL) fluids and whole lungs are collected to analyze lung permeability indexes **(B)**, measurement of pulmonary myeloperoxidase (MPO) contents **(C)**, total white blood cells **(D)**, and neutrophils counts **(E)** in BAL fluids, and levels of TNF-α **(F)**, MCP-1 **(G)**, MIP-1α **(H)**, and MIP-2 **(I)** in BAL fluids, respectively. Data are expressed as means ± S. E. M. N=3 for α-BSA-treated mice, N=5 for Ad-GFP+IgG-IC group, and N=6 for Ad-PPARγ+IgG-IC group. *, ** and *** indicate statistically significant difference—*p* < 0.05, *p* < 0.01, and *p* < 0.001, respectively.

We then investigate the role of PPARγ in IgG-IC-initiated acute lung injury which is reflected by lung permeability, and MPO activity—an indicator of neutrophil accumulation. Firstly, we overexpress PPARγ in the mouse pulmonary tissue by using adenovirus as the vector (24), and find that Ad-PPARγ greatly increases PPARγ expression even at a dose of 1 × 10^8^ PFU (plaque forming unit) (**Figure 1A**). Importantly, mice receive intratracheal treatment of 1 × 10^8^ PFU of adenoviral vectors do not show any signs of acute lung injury (**Figure S2**). Therefore, in the following studies, 1 × 10^8^ PFU of Ad-PPARγ is applied to the animal experiments. As shown in **Figure 1B**, when compared with the control group, lung permeability index is increased by more than 2.5 folds after IgG-IC stimulation, which is almost reduced to basal level by ectopic expression of PPARγ. As expected, the increase in the pulmonary MPO activity caused by IgG-IC treatment is also significantly inhibited by PPARγ overexpression (**Figure 1C**).

Recruitment of white blood cells, especially neutrophils, into alveolar spaces plays a critical role in acute lung injury. We observe that influxes of total leukocytes and neutrophils into alveolar compartments are greatly induced by IgG-IC treatment (**Figures 1D, E**). However, 4 h after initiation of IgG-IC-induced acute lung injury, the numbers of total white blood cells and neutrophils recovered from BAL fluids of Ad-PPARγ mice are obviously reduced as compared with the control (**Figures 1D, E**).

We further elucidate the influence of PPARγ on expressions of pro-inflammatory mediators that are pivotal initiators of acute lung injury. We find that upon IgG-IC stimulation, expressions of TNF-α, MCP-1, MIP-1α and MIP-2 are dramatically induced in BAL fluids (**Figures 1F–I**). However, ectopic expression of PPARγ in the lung decreases IgG-IC-stimulated expressions of TNF-α, MCP-1, MIP-1α and MIP-2 by 43%, 67%, 36%, and 43%, respectively (**Figures 1F–I**).

To further elucidate the important role of PPARγ in IgG-IC-induced acute lung injury, chemical reagent GW9662—an antagonist of PPARγ, is used to block PPARγ activation. As shown in **Figure S3**, IgG-IC-induced elevation of acute lung injury-related indicators including lung permeability, MPO activity, alveolar recruitment of leukocytes, especially neutrophils, and expressions of pro-inflammatory mediators are further exacerbated in mice receiving GW9662 treatment. However, acute lung injury induced by IgG-IC is alleviated by PPARγ agonist—ROSI (**Figure S4**)

### Inflammation Is Relieved by PPARγ in IgG-IC-Treated Macrophages

**Figure 2:**
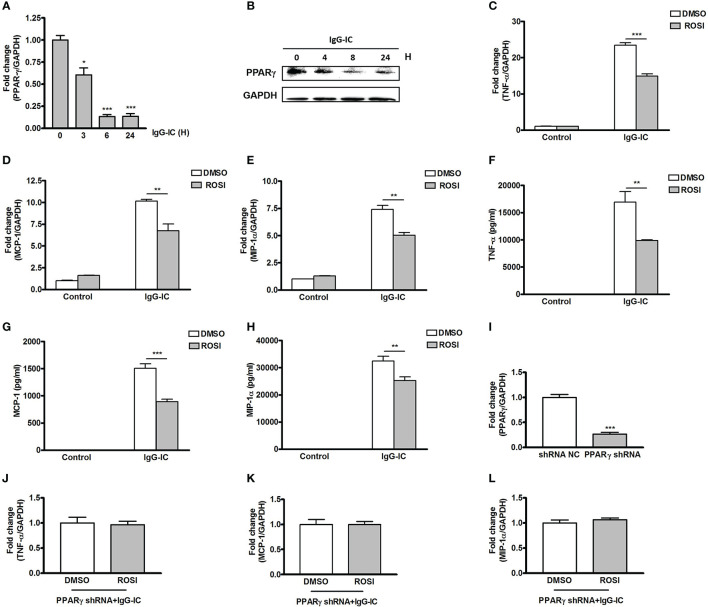
Peroxisome proliferator-activated receptor (PPAR)γ decreases IgG immune complex (IgG-IC)-stimulated inflammatory response in macrophages. RAW264.7 cells are treated with 100 μg/ml of IgG-IC for different time points. Then RNAs and proteins are extracted, and qPCR **(A)** and Western blot **(B)** are performed to verify PPARγ generation at mRNA and protein levels, respectively. RAW264.7 cells are pre-treated with DMSO or 10 μM Rosiglitazon–ROSI. One hour later, the cells are further treated with IgG-IC. Then total cellular RNAs are isolated 4 h later, and cell-free supernatants are harvested 8 h later. TNF-α **(C, F)**, MCP-1 **(D, G)**, and MIP-1α **(E, H)** expressions are examined at both RNA and protein levels. **(I)** RAW264.7 cells are infected by control or PPARγ shRNA lentiviral particles at a MOI of 30. Seventy-two hours later, the cells are treated with 3 μg/ml of puromycin. The survival cells are selected, and the knockdown efficiency is confirmed by qPCR. RAW264.7 cells expressing PPARγ shRNA are treated with DMSO or 10 μM ROSI for 1 h. Then the cells are treated with IgG-IC for 4 h, which is followed by measurement of TNF-α **(J)**, MCP-1 **(K)**, and MIP-1α **(L)** expressions. Data are expressed as means ± S. E. M. (N=3 for qPCR, and N=6 for ELISA). *, ** and *** indicate statistically significant difference—*p* < 0.05, *p* < 0.01, and *p* < 0.001, respectively.

During IgG-IC-induced acute lung injury, macrophage is one of the most important positive regulators of the severity of tissue damage. Therefore, the role of PPARγ in IgG-IC-induced inflammatory response in macrophages is analyzed. Firstly, we investigate whether PPARγ expression in macrophages is affected by IgG-IC at mRNA level. As shown in **Figure 2A**, PPARγ expression in macrophages is decreased in a time-dependent manner within 6 h after onset of IgG-IC treatment. Even after 24 h of IgG-IC stimulation, PPARγ maintains at low level (**Figure 2A**). Furthermore, the above qPCR results are consistent with the data obtained from Western blot analysis (**Figure 2B**).

We then determine the effect of PPARγ on IgG-IC-stimulated pro-inflammatory mediators’ generation by using PPARγ agonist Rosiglitasone (ROSI). We find that expressions of TNF-α, MCP-1 and MIP-1α at mRNA level are increased by 22-, 10-, and seven-folds, respectively in macrophages treated with IgG-IC (**Figures 2C–E**). ROSI treatment causes IgG-IC-mediated productions of TNF-α, MCP-1, and MIP-1α to decrease by 36%, 34%, and 32%, respectively (**Figures 2C–E**). We next verify the influence of ROSI on IgG-IC-initiated inflammatory reaction at protein level, and observe that the data obtained from ELISA are consistent with the qPCR results (**Figures 2F–H**).

To exclude the off-target effect of ROSI, we silence the expression of PPARγ in macrophages. As shown in **Figure 2I**, macrophages treated with PPARγ shRNA show a 73% decrease in PPARγ production when compared with the control cells. Of note, IgG-IC-mediated productions of TNF-α, MCP-1 and MIP-1α are no longer reduced by ROSI in macrophages expressing PPARγ specific shRNA (**Figures 2J–L**), indicating that ROSI negatively regulates IgG-IC-induced expressions of cytokine and chemokine *via* activating PPARγ.

### Egr-1 Expression Is Reduced by PPARγ in Macrophages

**Figure 3:**
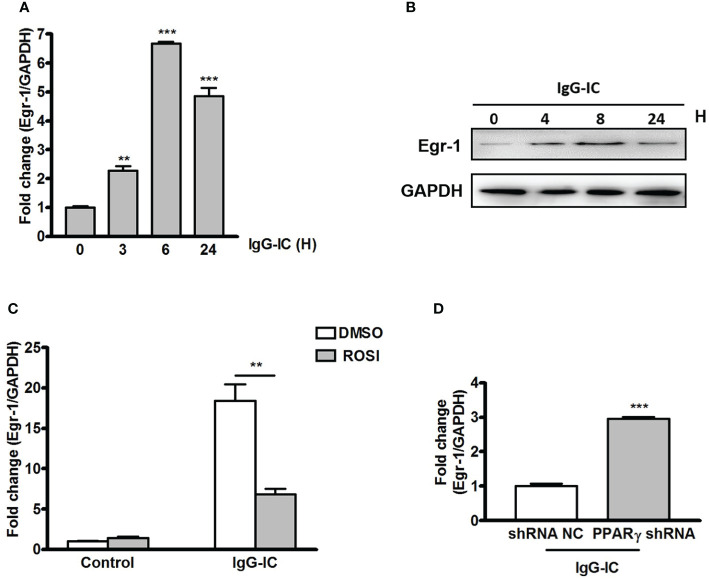
Early growth response protein 1 (Egr-1) production is negatively regulated by peroxisome proliferator-activated receptor (PPAR)γ in macrophages. RAW264.7 cells are challenged by IgG immune complex (IgG-IC) for distinct time points. Then RNAs and proteins are extracted, and qPCR **(A)** and Western blot **(B)** are conducted to analyze Egr-1 expression. **(C)** RAW264.7 cells are pre-treated with DMSO or 10 μM Rosiglitazone (ROSI) for 1 h. Then the cells are incubated with IgG-IC for 3 h, and Egr-1 expression is examined. **(D)** RAW264.7 cells expressing control or PPARγ shRNA are treated with IgG-IC for 3 h, which is followed by measurement of Egr-1 generation. Data are expressed as means ± S. E. M. (N=3). ** and *** indicate statistically significant difference—*p* < 0.01, and *p* < 0.001, respectively.

To probe the mechanism by which PPARγ decreases IgG-IC-induced inflammatory response, we tend to find out a bridge molecule linking PPARγ with pro-inflammatory mediators. Time course experiment shows that Egr-1 mRNA level peaks after 6 h of IgG-IC stimulation, and then declines progressively (**Figure 3A**), which is consistent with Western blot results (**Figure 3B**). More importantly, we find that in the presence of IgG-IC, Egr-1 expression in ROSI-treated macrophages is only 37% of the control (**Figure 3C**). On the contrary, IgG-IC-stimulated Egr-1 expression is increased by approximately twofolds with downregulation of PPARγ production (**Figure 3D**), suggesting that Egr-1 might be the possible molecule connecting PPARγ with pro-inflammatory mediators.

### Egr-1 Amplifies IgG-IC-Induced Expressions of Pro-Inflammatory Mediators in Macrophages by Binding to Their Promoter Regions

**Figure 4:**
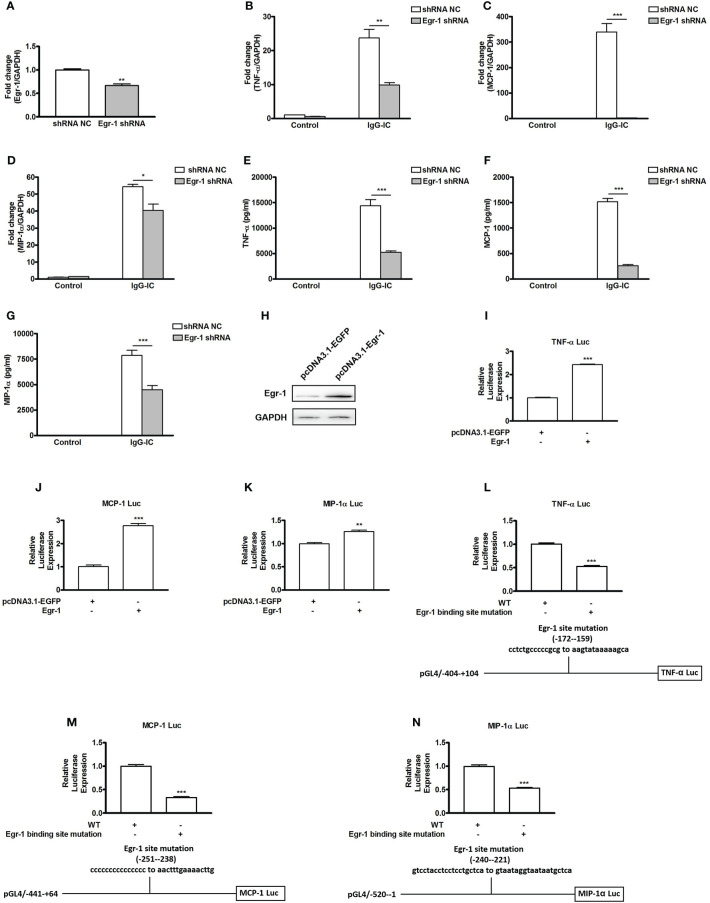
Early growth response protein 1 (Egr-1) positively regulates IgG immune complex (IgG-IC)-triggered expressions of TNF-α, MCP-1, and MIP-1α in macrophages by binding to their promoter regions. **(A)** RAW264.7 cells are infected by control or Egr-1 shRNA lentiviral particles at a MOI of 30. Seventy-two hours later, the cells are treated with 3 μg/ml of puromycin. The survival cells are selected, and the knockdown efficiency is confirmed by qPCR. RAW264.7 cells expressing control or Egr-1 shRNA are treated with IgG-IC. Then RNAs and cell-free supernatants are harvested 4 and 8 h later, respectively. qPCR and ELISA are performed to analyze TNF-α **(B, E)**, MCP-1 **(C, F)**, and MIP-1α **(D, G)** expressions. **(H)** Egr-1-overexpressing plasmids are constructed, and confirmed in HEK293 cells by Western blot. **(I–N)** HEK293 cells are transfected with the indicated plasmids. Twenty-four hours later, the cells are lysed, and the lysates are subjected to luciferase assays. The detailed information about the luciferase-expressing constructs is indicated in the figures. The data are calculated by dividing the value of the indicated promoter-driven firefly luciferase by the corresponding value of thymidine kinase promoter-driven renilla luciferase, and then set to 1 in control cells. Data are expressed as means ± S. E. M. (N=3 for qPCR and luciferase assays, and N=6 for ELISA). *, ** and *** indicate statistically significant difference—*p* < 0.05, *p* < 0.01, and *p* < 0.001, respectively.

Egr-1 expression is controlled by PPARγ, but its effect on IgG-IC-triggered pro-inflammatory mediators’ expressions remains elusive. Therefore, we first downregulate Egr-1 expression in macrophages by using lentivirus expressing Egr-1 specific shRNA (**Figure 4A**). In the presence of IgG-IC, TNF-α, MCP-1, and MIP-1α mRNA levels are increased by more than 20, 300, and 50 times, respectively (**Figures 4B–D**). Egr-1 shRNA treatment reduces the expressions of the above inflammatory mediators to 41%, 1%, and 74% of their respective controls (**Figures 4B–D**). Furthermore, the suppressive role of Egr-1 shRNA in inflammation is also confirmed at protein level in macrophages challenged by IgG-IC (**Figures 4E–G**).

To reveal the mechanism involved in Egr-1 regulation of pro-inflammatory mediators’ generation, we firstly construct Egr-1 overexpression plasmid (**Figure 4H**). Then we investigate if productions of pro-inflammatory mediators are influenced by ectopic expression of Egr-1. As shown in **Figures 4I–K**, TNF-α, MCP-1, and MIP-1α promoter-driven luciferase activities are increased by Egr-1 to 242%, 277%, and 126% of their respective controls. According to software prediction (JASPAR), there are potential Egr-1 binding sites in the promoter regions of the above pro-inflammatory mediators. Thus, we further introduce mutations to the Egr-1 sites of the above inflammatory mediators’ promoters, and find that Egr-1 site mutation causes TNF-α, MCP-1 and MIP-1α promoter-driven luciferase activities to decrease by 47%, 67%, and 47%, respectively (**Figures 4L–N**).

### During IgG-IC-Induced Acute Lung Injury, Egr-1 Expression Is Reduced by PPARγ

**Figure 5:**
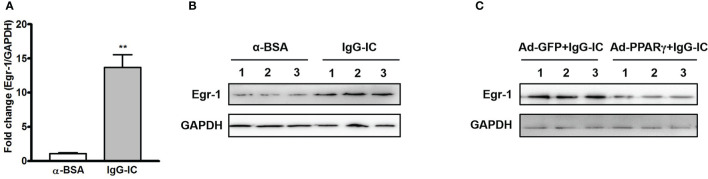
IgG immune complex (IgG-IC)-mediated pulmonary early growth response protein 1 (Egr-1) expression is inhibited by ectopic production of peroxisome proliferator-activated receptor (PPAR)γ. Four hours after pulmonary deposition of IgG-IC, lungs are harvested. The tissue RNAs and proteins are isolated separately. Then the Egr-1 expression is examined by qPCR **(A)** and Western blot **(B)**, respectively. **(C)** Mice are treated with airway injection of Ad-GFP or Ad-PPARγ at a dose of 1 × 10^8^ PFU. Seventy-two hours later, the mice are intratracheally challenged by IgG-IC for 4 h. Then the lungs are harvested, and proteins are extracted for Western blot analysis by using antibodies against Egr-1 and GAPDH, respectively. Data are expressed as means ± S. E. M. (For qPCR, N=3 for α-BSA treated mice, and N=5 for IgG-IC-treated mice; N=3 for Western blot). ** indicates statistically significant difference—*p* < 0.01.

To clarify the effect of PPARγ on Egr-1 expression *in vivo*, we first examine the production of Egr-1 during IgG-IC-induced acute lung injury. As shown in **Figure 5A**, Egr-1 mRNA level in the lung tissue is increased to 13.7-folds of the control group. Furthermore, IgG-IC also stimulates an obvious increase in Egr-1 protein expression when compared with the control treatment (**Figure 5B**). More importantly, during IgG-IC-induced acute lung injury, we find that mice receiving airway administration of Ad-PPARγ show a great reduction in Egr-1 generation when compared with their littermates treated by Ad-GFP (**Figure 5C**).

### Egr-1 Aggravates IgG-IC-Induced Acute Lung Injury Through Amplifying Expressions of Pro-Inflammatory Mediators

**Figure 6:**
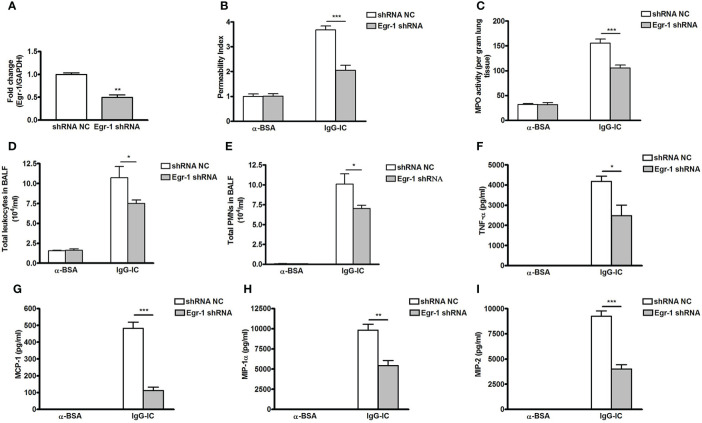
Downregulating early growth response protein 1 (Egr-1) expression attenuates IgG immune complex (IgG-IC)-mediated acute lung injury. **(A)** Lentiviruses (3 × 10^7^ TU—transduction unit) expressing control or Egr-1 shRNA are injected into mouse lungs through airways. Five days later, the lungs are harvested, and RNAs are extracted, which is followed by verification of Egr-1 expression by qPCR. Mice infected by control or Egr-1 shRNA lentiviral particles are intratracheally treated by IgG-IC for 4 h. Then bronchoalveolar lavage (BAL) fluids and whole lungs are harvested. Lung permeability index **(B)**, and measurement of pulmonary myeloperoxidase (MPO) activities **(C)** in whole lungs are measured. In addition, influxes of total leukocytes **(D)** and neutrophils **(E)** into alveolar spaces are counted. Also, productions of TNF-α **(F)**, MCP-1 **(G)**, MIP-1α **(H)**, and MIP-2 **(I)** in BAL fluids are assayed. Data are expressed as means ± S. E. M. (N=3 for qPCR; For ELISA, N=3 for α-BSA-treated mice, N=5 for shRNA NC+IgG-IC group, and N=6 for Egr-1 shRNA+IgG-IC group). *, ** and *** indicate statistically significant difference—*p* < 0.05, *p* < 0.01, and *p* < 0.001, respectively.

Next, we analyze the role of Egr-1 in IgG-IC-induced acute lung injury by downregulating its expression with the lentiviral vector (25) (**Figure 6A**). Of note, lentiviral vector itself could not stimulate acute lung injury (**Figure S5**). We find that after airway deposition of IgG-IC, mice treated with Egr-1 shRNA display reduced tissue damage signs including lung permeability and MPO activity as compared with the control mice receiving shRNA NC treatment (**Figures 6B, C**). Additionally, IgG-IC-induced recruitment of white blood cells including neutrophils into the alveolar compartments is significantly relieved by downregulating Egr-1 expression in the lungs (**Figures 6D, E**).

Productions of pro-inflammatory mediators promote acute lung injury. So we examine the effect of Egr-1 on expressions of cytokine and chemokine. As shown in **Figures 6F–I**, IgG-IC stimulation dramatically elevates TNF-α, MCP-1, MIP-1α, and MIP-2 expressions in BAL fluids, which are significantly decreased with downregulation of Egr-1 generation in the lung tissue.

## Discussion

Due to lacking of specific therapy methods in clinic, the mortality rates of ALI/ARDS in the United States can be as high as 60%, despite the recent development of protective lung ventilation strategies (26, 27). Thus, we should further investigate the molecular mechanisms of ALI/ARDS to promote the development of specific therapies. In the present study, we utilize IgG-IC-induced acute lung injury model to reveal the underlying mechanism of acute lung injury. We have proved that IgG-IC treatment causes an obviously acute inflammatory response, characterized by the infiltration of neutrophils into the pulmonary tissue, and the elevated expressions of cytokine and chemokine, which are similar to clinical symptoms. Furthermore, we find that PPARγ-Egr-1-pro-inflammatory mediators axis is involved in IgG-IC-triggered pulmonary damage, and intervention of the axis might be a useful strategy for treatment of patients suffering from ALI/ARDS.

After intrapulmonary deposition of IgG-IC, local macrophages expressing high levels of Fcγ receptors, but not alveolar epithelial cells first perceive the stimulation. Then early response cytokines including TNF-α and IL-1β are released from the macrophages, which are crucial for stimulating lung endothelial cells to express adhesion molecules (28, 29). Moreover, the early response cytokines promote chemokine secretion in the lung tissue (30–32). Finally, pulmonary injury and massive influx of neutrophils into the lung tissue are induced by the joint action of a variety of inflammation-related mediators including cytokines, chemokines and adhesion molecules. Actually, all the above processes are controlled by the network composed of various transcription factors. There are reports that the DNA binding activities of NF-κB and AP-1 are increased during IgG-IC-induced acute lung injury (31, 33). Both transcription factors can directly bind to a broad spectrum of pro-inflammatory mediators’ promoter regions, and increase their productions. In addition, our findings demonstrate that both C/EBPβ and C/EBPδ, which belong to C/EBP family members, can increase IgG-IC-induced inflammation *via* association with the C/EBP elements in the promoter regions of inflammatory genes (34, 35). In the present study, we find that PPARγ expression is significantly downregulated following IgG-IC treatment. However, as an important transcription factor, the role of PPARγ in IgG-IC-initiated inflammation has not been determined.

In the current paper, we find that PPARγ inhibits IgG-IC-induced TNF-α, MCP-1 and MIP-1α productions *in vitro* and *in vivo*. Previous studies have proved that PPARγ activation alleviates postoperative ileus-related gene expression through suppression of Egr-1 production (36). Also, Rosiglitazone-mediated PPARγ activation inhibits Egr-1 generation, leading to suppression of bleomycin-induced scleroderma and profibrotic responses (37). Thus, during IgG-IC-induced inflammatory responses, Egr-1 might be the potential bridge molecule linking PPARγ with pro-inflammatory mediators’ productions. We find that Egr-1 expressions in macrophages and lungs are greatly upregulated by IgG-IC treatment. In addition, we observe that activation of PPARγ decreases Egr-1 expression, while lowing PPARγ level increases its generation, indicating that Egr-1 is negatively regulated by PPARγ. Furthermore, we demonstrate that during IgG-IC-induced acute inflammatory response, expressions of pro-inflammatory mediators are positively regulated by Egr-1. Thus, during IgG-IC-induced acute lung injury, PPARγ can negatively regulate IgG-IC-induced inflammation through an indirect way, which is similar to the method used by STAT3 (38). Of note, during IgG-IC-mediated acute lung injury, whether there are other PPARγ-regulated transcription factors that directly control inflammation responses remains an open question.

Egr-1, a critical transcription factor, belongs to the EGR family of Cys2His2-type zinc finger proteins. It is composed of transactivation and inhibitory domains, as well as three DNA-binding zinc finger domains which interact with GC-rich regions in the promoters of target genes (39). Its roles in lung diseases, especially in chronic lung diseases have been widely reported. Lee et al. find that Egr-1-mediated cell apoptosis is crucial for TGF-β-elicited lung fibrosis (40), indicating that Egr-1 plays a detrimental role in the disease. In contrast, Kramer et al. report that the deficiency of Egr-1 obviously exacerbates TGF-α-induced pulmonary fibrosis, which is independent of the tissue inflammatory response (41), which manifests the protective effect of Egr-1 in the disease. The above contradictory phenomena might be explained by the distinct stimulus used in the model. Therefore, the role of Egr-1 is complicated, and should be discussed in specific contexts. In the present paper, we observe that Egr-1 accelerates inflammation induced by IgG-IC through association with the promoter regions of pro-inflammatory mediators including TNF-α, MCP-1 and MIP-1α. Our previous data have demonstrated that the activities of both p38 MAPK and ERK1/2 are elicited, and are essential for cytokine/chemokine expressions during IgG-IC-induced inflammation (34). Others find that p38 MAPK activation is required for Egr-1-dependent expressions of pro-inflammatory mediators (42). Moreover, there are data that the IL-33-ERK/JNK/p38/Egr-1/TSLP (thymic stromal lymphopoietin) axis is involved in allergic skin Th2 inflammation (43). Thus, we speculate that there may be crosstalk between MAPK signaling pathway and PPARγ-Egr-1 axis during inflammatory reactions triggered by IgG-IC. However, this remains an open question, which should be supported by more studies. Additionally, the role of the inhibitory domain of Egr-1 in IgG-IC-induced inflammation should also be examined in the future.

On the basis of our present data, we conclude that PPARγ protects against the development of IgG-IC-induced acute lung injury through the reduction of inflammatory responses. This protection afforded by PPARγ features reduced cytokine/chemokine expressions and accumulation of neutrophils in the lung tissue. Furthermore, the anti-inflammatory effects of PPARγ after intrapulmonary deposition of IgG-IC are mediated by inhibiting generation of Egr-1, which can bind to the promoter regions of pro-inflammatory mediators including TNF-α, MCP-1, and MIP-1α. Therefore, PPARγ activation might represent a promising therapeutic option for IgG-IC-induced acute lung injury. Though PPARγ agonist Rosiglitazone has been used for treatment of type 2 diabetes in clinic, whether the protective effects of PPARγ detected in the animal model could be extrapolated to the clinical application is subject to the future clinical trials.

## Data Availability Statement

The raw data supporting the conclusions of this article will be made available by the authors, without undue reservation.

## Ethics Statement

The animal study was reviewed and approved by Animal Experimental Ethical Committee of Southeast University.

## Author Contributions

CY, JC, YD, ZZ, BL, CD, and DY performed experiments. CY, QZ, and XW designed the experiments. CY and XW drafted the article. All authors contributed to the article and approved the submitted version.

## Funding

This project is supported by the National Natural Science Foundation of China (grant numbers 81971858 and 31400751), Fundamental Research Funds for the Central Universities (grant number 3224002002C1), and the Scientific and Technological Project of Jintan District (grant number KJ201923).

## Conflict of Interest

The authors declare that the research was conducted in the absence of any commercial or financial relationships that could be construed as a potential conflict of interest.
