# Supplementary material for: The Crucial Role of PPARγ-Egr-1-Pro-Inflammatory Mediators Axis in IgG Immune Complex-Induced Acute Lung Injury
Source: Front Immunol. 2021 Feb 25;12:634889. doi: 10.3389/fimmu.2021.634889 (PMC7947684; doi:10.3389/fimmu.2021.634889)

**Supplementary figure legends**

**Fig. S1 PPARγ expression in lungs is downregulated after airway deposition of IgG-IC.** Mice receive intratracheal deposition of IgG-IC, and lungs are harvested four hours later. Then RNAs and proteins are extracted from the tissues. qPCR (A) and Western blot (B) are conducted to analyze PPARγ expression at mRNA level and protein level, respectively. Data are expressed as means ± S. E. M.. For qPCR, N=3 for α-BSA-treated mice, and N=4 for IgG-IC-treated littermates. For Western blot, N=3. * indicates statistically significant difference—*p* < 0.05.

**Fig. S2 Ectopic expression of adenoviral vectors in the lung cannot induce acute lung injury.** A. PBS or 1 × 10^8^ PFU of Ad-GFP is injected into lungs *via* airways. 3 days later, acute lung injury is induced by treating the mice with IgG-IC. 4 hours later, BAL fluids and whole lungs are collected to analyze lung permeability indexes (A), and MPO contents (B), respectively. Data are expressed as means ± S. E. M.. N=3 for α-BSA-treated mice, N=6 for IgG-IC-treated groups.

**Fig. S3 PPARγ antagonist treatment elevates IgG-IC-induced acute lung injury.** Mice receive intraperitoneal administration of DMSO or GW9662 (5 mg/kg). 30 min later, acute lung injury is stimulated by intratracheal deposition of IgG-IC. 4 hours later, BAL fluids and whole lungs are collected to analyze lung permeability indexes (A), MPO contents (B), and total white blood cells (C) and neutrophils counts (D) in BAL fluids, and levels of TNF-α (E), MCP-1 (F), MIP-1α (G) and MIP-2 (H) in BAL fluids, respectively. Data are expressed as means ± S. E. M.. N=3 for α-BSA-treated mice, N=5 for DMSO+IgG-IC group, and N=6 for GW9662+IgG-IC group. ** and *** indicate statistically significant difference—p < 0.01, and p < 0.001, respectively.

**Fig. S4 PPARγ agonist treatment decreases IgG-IC-induced acute lung injury.** Mice receive intraperitoneal administration of DMSO or ROSI (10 mg/kg). 30 min later, acute lung injury is stimulated by intratracheal deposition of IgG-IC. 4 hours later, BAL fluids and whole lungs are collected to analyze lung permeability indexes (A), MPO contents (B), and total white blood cells (C) and neutrophils counts (D) in BAL fluids, respectively. Data are expressed as means ± S. E. M.. N=3 for α-BSA-treated mice, and N=6 for IgG-IC-treated groups. *, ** and *** indicate statistically significant difference—p < 0.05, p < 0.01, and p < 0.001, respectively.

**Fig. S5 Ectopic expression of lentiviral vectors in the lung cannot induce acute lung injury.** A. PBS or lentivirus (3 × 10^7^ TU) expressing control shRNA is injected into mouse lungs through airways. 5 days later, mice are intratracheally treated by IgG-IC for 4 hours. Then BAL fluids and whole lungs are harvested to analyze lung permeability indexes (A), and MPO contents (B), respectively. Data are expressed as means ± S. E. M.. N=3 for α-BSA-treated mice, N=6 for IgG-IC-treated groups.

**Supplementary Fig. 1**

**
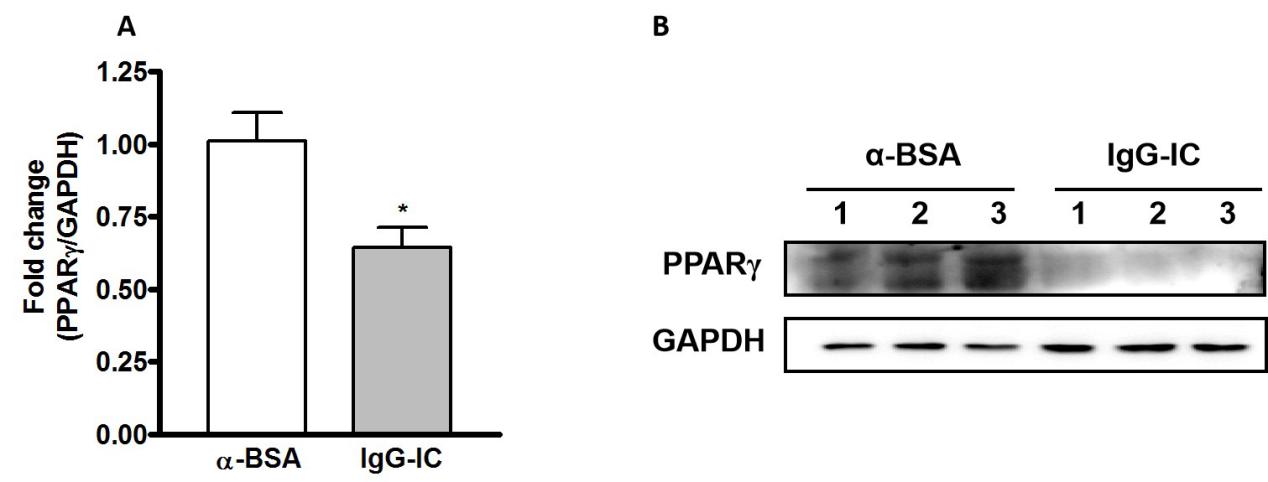
**

**Supplementary Fig. 2**

**
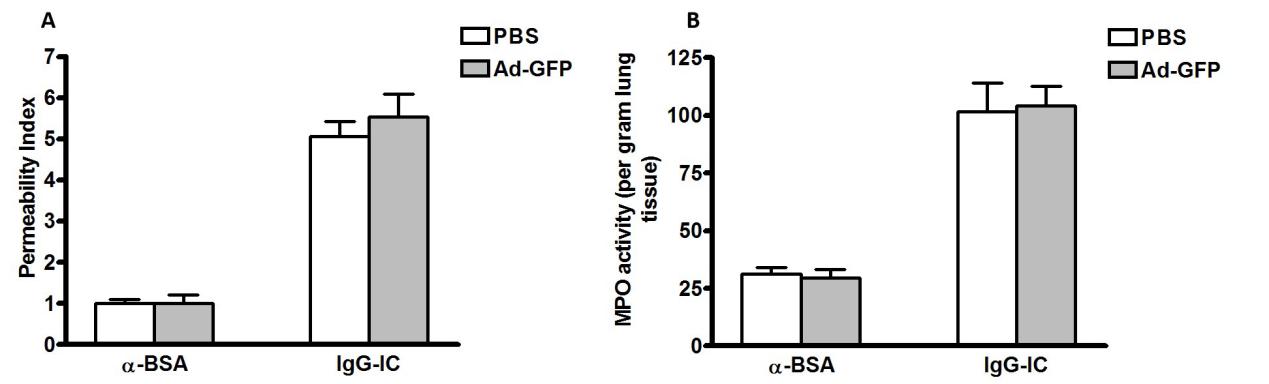
**

**Supplementary Fig. 3**


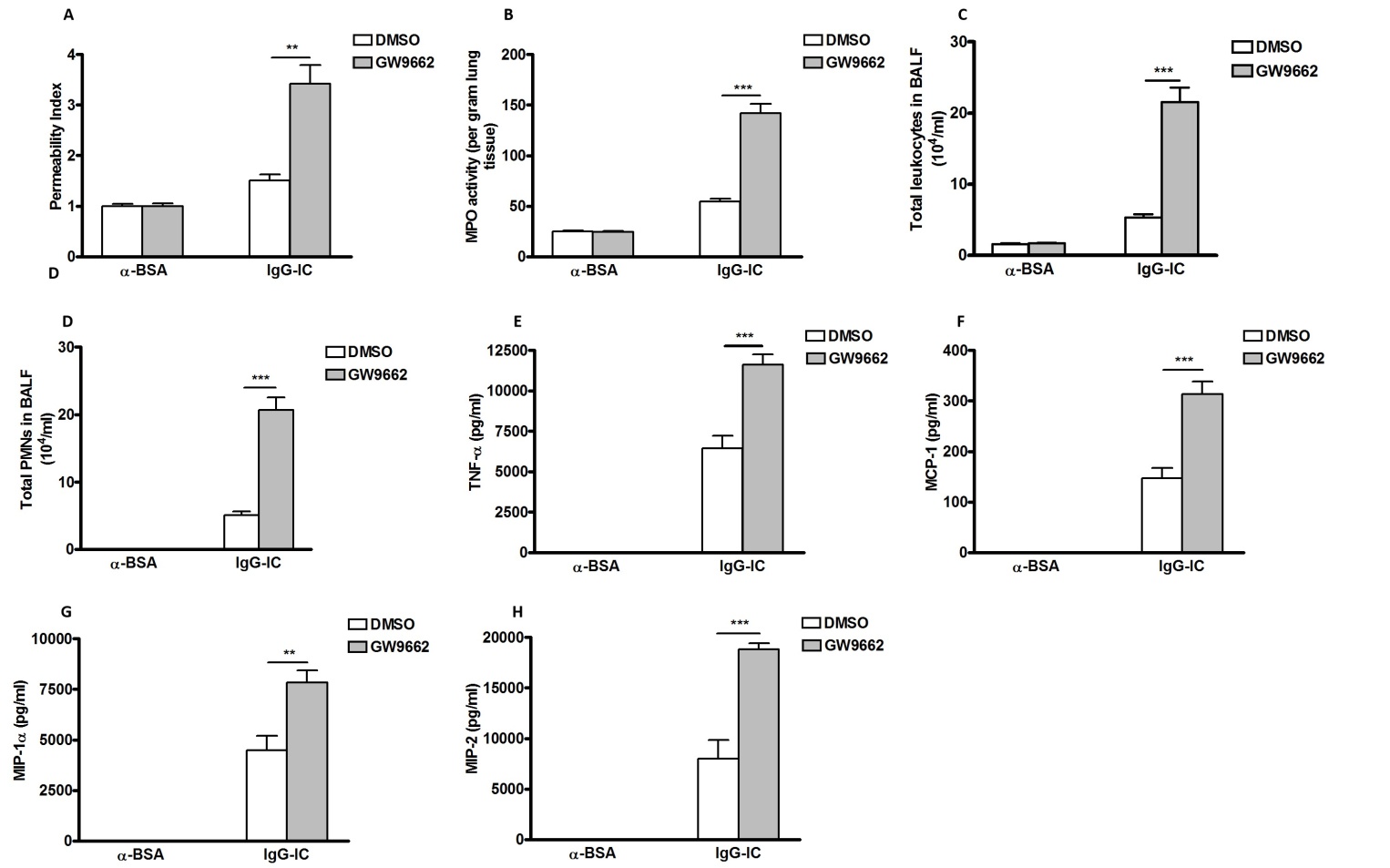


**Supplementary Fig. 4**


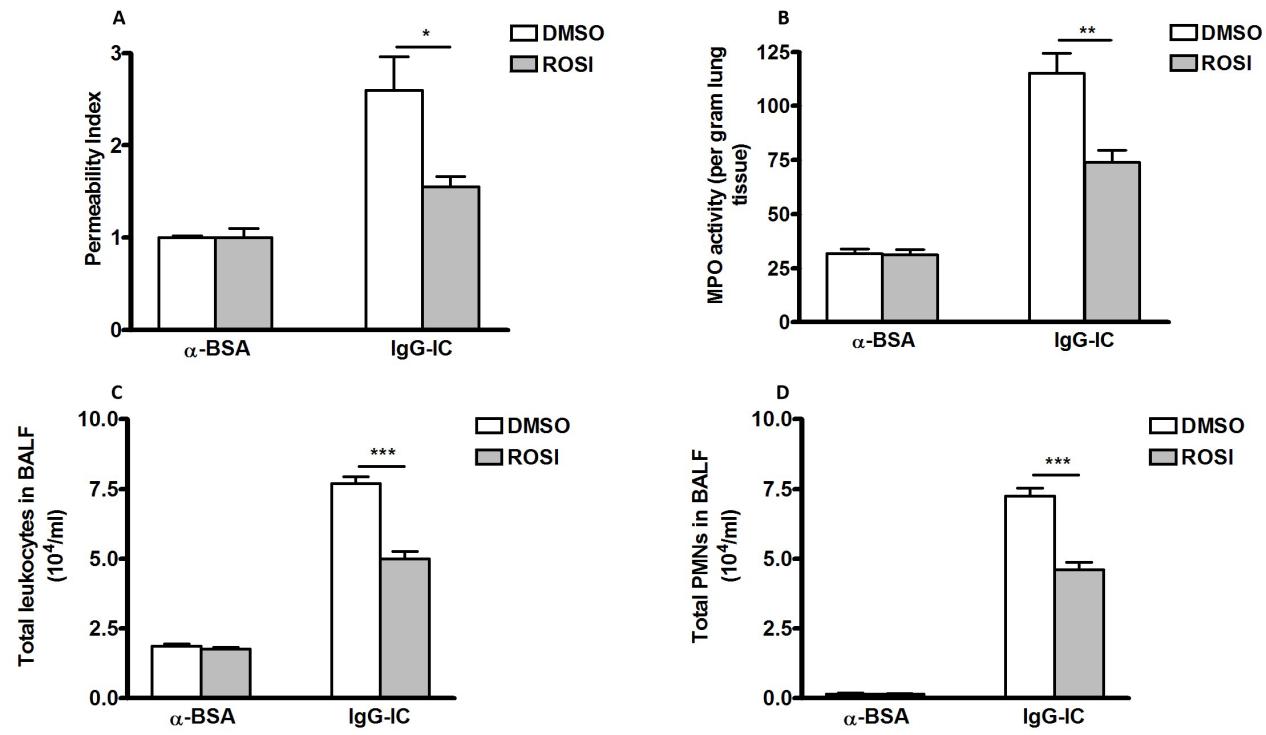


**Supplementary Fig. 5**


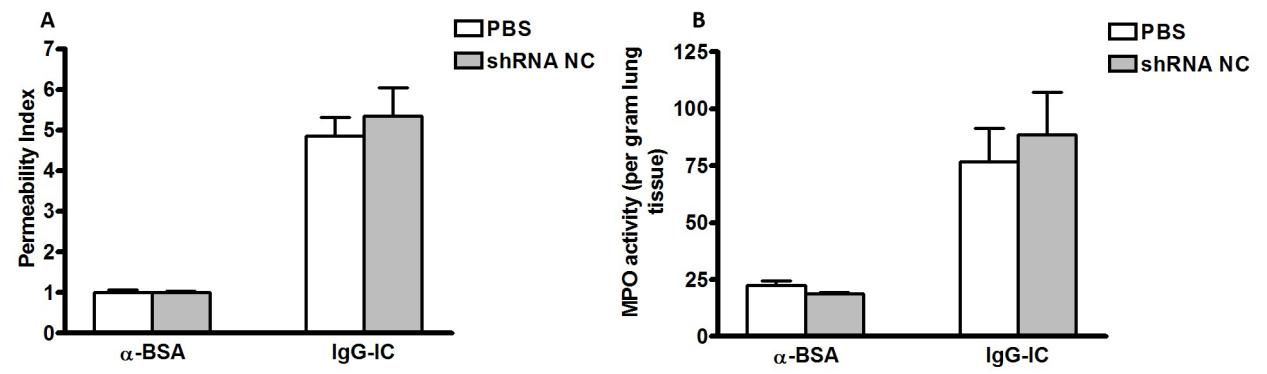

Supplement: Supplementary file 1 [file DataSheet_1.docx]
